# Volumetric analysis of maxillary sinuses for gender determination: Manual vs.3D segmentation

**DOI:** 10.6026/973206300200882

**Published:** 2024-08-31

**Authors:** Naveen Kumar Shetty, Bhavika Pol Vhatkar, Shweta Rode, Abhishek Mukherjee

**Affiliations:** 1 Department of Oral Medicine & Radiology, School of Dentistry, DY Patil (deemed to be university), Navi Mumbai, India

**Keywords:** 3D segmentation, Manual Calculation, Maxillary Sinus Volume, Volumetric analysis

## Abstract

The effectiveness of manual calculations versus 3D segmentation techniques in volumetric analysis of maxillary sinuses for gender determination is of interest. Maxillary sinuses, which vary anatomically due to factors like age, ethnicity, and gender, are crucial in forensic and anthropological contexts. Traditional methods, relying on two-dimensional imaging, are often time-consuming and prone to errors, whereas 3D segmentation offers a more precise and efficient approach. This research evaluates both methods in terms of reliability, accuracy, and practical use, potentially influencing their application in clinical and forensic settings. The findings may also enhance understanding of anatomical variations in maxillary sinuses across populations, contributing to more accurate gender determination.

## Background:

The pyramid shaped maxillary sinus possesses the maximum dimensions amongst all the paranasal sinuses. It aids in the mucous drainage and air flow of the same [1]. It is located on the lateral wall of the nasal cavity and has several well defined borders [2]. Average volume of a healthy maxillary sinus at maturity ranges between 15-20,000 cubic mm [3,4]. The volume of maxillary sinuses can also be used as gender identification tools in forensic odontology [5]. Various studies have also indicated a correlation between maxillary sinus volume & anatomic variations such as nasal septal deviation & concha bullosa [6].These variations in turn lead to various maxillary sinus pathologies such as sinusitis & antro-choanal polyps [7]. It has also been well established in literature about the larger volumes of maxillary sinuses in males as compared to females [8]. The estimation of maxillary sinus volume(MSV) can be ascertained via manual estimation as well as using highly precise segmentative software [9]. The manual calculation has been successfully attempted mostly using the geometric formula for volume calculation of a pyramid taking into account anatomically similar shape of the sinus while software's with segmentation algorithms such as ITK-SNAP(various versions) & Advantage workstation have been instrumental in deducing MSV values from CT,CBCT & MRI scans. The gold standard for evaluation in multiplanar imaging projections is CT [10]. Other maxillary sinus imaging modalities include Water's view, Caldwell view, lateral view, basal and submento vertex radiographs are the most commonly used conventional radiographs for evaluation of paranasal sinus pathologies. CBCT has recently become a popular multiplanar sinus imaging modality [11,12]. Therefore, it is of interest to report the effectiveness of different methods for maxillary sinus volumetric analysis and their potential role in gender prediction.

## Materials & Methods:

"The present retrospective study was approved by the Institutional Review and Ethics Board of DY Patil University School of Dentistry Navi Mumbai" on 26/2/2024 & the reference number allotted was IREB/2024/OMR/01.

## Sample size estimation & study design:

The sample size estimation for the current retrospective study was done using "G power software version 3.1.9.6 by the Franz Faul University Kiel.

The sample size was estimated to be around 80 for each group (males & females) i.e. 160 CBCT scans. Subsequently CBCT scan data was taken for 160 patients (80 males & 80 females) in the age group of 20-72 years were obtained from archives from March 2022 till September 2023. CBCT scans were taken on Kodak Carestream CS9600 full volume CBCT unit with FOV 16X10 cm. We included CBCT scans of 160 patients (80 females & 80 males) in the age group 20 -72 years with healthy sinuses were analyzed (320 sinuses in total). We excluded CBCT scans that showed diffuse opacifications in either sinus such as sinusitis or polypoid structures which would have affected calculation of width, height or length & therefore volume. Scans with maxillary sinus wall fractures were also excluded.

## Manual maxillary sinus volume (MSV) calculation:

Manual volume calculation of each maxillary sinus was done using the proven geometric formula for volume calculation of a pyramid as the maxillary sinus is a pyramidal structure LxWxHx0.52 where L is the antero-posterior sinus extent on sagittal section, W is the maximum medio-lateral sinus diameter on coronal section & H is the maximum sinus height on supero-inferior dimension on coronal section. Measurements for manual volume calculation were given using pre-installed measuring tool on CS Imaging software.

## Automatic Maxillary Sinus Volume (MSV) calculation:

Automatic sinus volume analysis was done using ITK SNAP version 4.2.0 segmentation software. The SNAKE contouring tool within the ITK-Snap application was first used to delineate the ROI (region of interest) Vis-a Vis the right & left maxillary sinuses respectively. Subsequently, Sky Blue was used as the active label pre-segmentation color for the right maxillary sinus for each patient while Bright Red was used as active label pre-segmentation colour for the left maxillary sinus respectively. Patients were divided into 2 groups (Manual & Automatic Calculation) respectively. Microsoft Excel (2007) was used for data entry in the current study and subsequently analyzed using the SPSS statistical software 23.0 Version. To calculate "intergroup comparison for the difference of mean scores between two independent groups, unpaired/independent t test was applied while Shapiro-Wilk tests were done for the purpose of data distribution. To ascertain the homogeneity of the variables, Levene's test was performed. Thereafter Discriminant analysis was applied for purpose of gender prediction.

## Results:

Among females the mean right MSV was 11755.9 + 4237 cubic mm in the manual method and 11747.86 + 3442 cubic mm in the automatic method. The mean left MSV was 11811.7 + 4176 cubic mm in the manual method and 11957.3 + 3278 cubic mm in the automatic method. The intergroup comparison between manual and automatic method was statistically non-significant for both left and right maxillary sinuses as seen in the Table 1 and Bar Graph 1 below:

Among the males the mean right MSV was 17304.5 +7418 cubic mm in the manual method and 16419.25 + 6659 cubic mm in the automatic method. The mean left MSV was 16283.87 + 4176 cubic mm in the manual method and 17010.62 + 3278 cubic mm in the automatic method. The intergroup comparison between manual and automatic method was statistically non-significant on both left and right side as shown in Table 2 & Bar graph 2 below

Among the females the mean right MSV was 11755.9 + 4237 cubic mm and among the males the same was 17304.5 + 7418 cubic mm. With respect to the left MSV, the mean volume among the females was 11811.7 + 4176 cubic mm and 16283.87 + 4176 cubic mm in the males. The intergroup comparison between males and female method was statistically highly significant for both left and right maxillary sinuses as noted in Table 3 & Bar Graph 3 below:

Among the females the mean right MSV was 11747.86 + 3442 cubic mm and among the males the same was 16419.25 + 6659 cubic mm. The mean left MSV among the females was 11957.3 + 3278 cubic mm and the same was 17010.62 + 3278 cubic mm in the males. The intergroup comparison between males and females method was statistically highly significant on both left and right side as denoted in Table 4 &Bar graph 4 below.

73.3% of the females were correctly predicted and 62.5% of the males were correctly predicted by the manual method. The overall accuracy of the manual method for gender prediction based on right maxillary sinus volume was 69.6%

80.0% of the females were correctly predicted and 62.5% of the males were correctly predicted by the manual method. The overall accuracy of the manual method for gender prediction based on left maxillary sinus volume was 73.9%.

73.3% of the females were correctly predicted and 50.0% of the males were correctly predicted by the automatic method. The overall accuracy of the automatic method for gender prediction based on right maxillary sinus volume was 65.2%.

83.3% of the females were correctly predicted and 56.2% of the males were correctly predicted by the automatic method. The overall accuracy of the automatic method for gender prediction based on left maxillary sinus volume was 73.9% as shown in Table 5.

## Discussion:

In our study, we observed differences between the maxillary sinus volume (MSV) values obtained through manual volumetric estimation and those derived from 3D segmentation. Specifically, for male patients, the MSV of the right maxillary sinus was found to be 0.9 cm^3^ larger using automatic analysis compared to manual calculations. For the left maxillary sinus, the values were nearly identical between the two methods for males. In contrast, the MSV values for both right and left maxillary sinuses in female patients were almost equivalent when comparing manual and automatic segmentation. Manual calculations were performed using the formula:

Volume = LxWxHx0.52, which is designed for pyramidal volumes.

This approach is consistent with the methods used by Sahlstrand-Johnson and Sharma in their studies. However, Mohlhenreich criticized linear measurements and mathematical formulae for their inaccuracies in MSV assessment, prompting us to also use ITK-SNAP version 4.2.0 for manual calculations. Przystanska *et al.* advocated using the pyramid formula for manual MSV calculations, suggesting that the maxillary sinus shape lies between a sphere and a pyramid. Their findings, which indicated greater reliability of manual estimations compared to software-generated results, contrast with our study's observation of similar values from both manual and automatic methods.

We recorded MSV values of 17.3 cm^3^ ± 7.4 cm^3^ for the right maxillary sinus in males via manual calculation, while automatic analysis yielded 16.4 cm^3^ ± 6.6 cm^3^. For the left maxillary sinus in males, manual calculations produced 16.3 cm^3^ ± 4.1 cm^3^, compared to 17 cm^3^ ± 3.2 cm^3^ from automatic analysis. These results align closely with those reported by Prabhat *et al.* (2016), who found right and left MSV values of 16.6 cm^3^ and 15 cm^3^, respectively, and Sahlstrand-Johnson *et al.* (2011), who reported a mean MSV of 15.7 cm^3^ ± 5.3 cm^3^ for males. However, Sahlstrand-Johnson used CT images, while our study employed CBCT. Gomes *et al.* reported higher MSV values of 19.9 cm^3^ and 19.8 cm^3^ for the right and left maxillary sinuses using ITK-SNAP version 3.0, compared to the values obtained with ITK-SNAP version 4.2.0 in our study.

For female patients, our study found MSV values of 11.75 cm^3^ ± 4.2 cm^3^ and 11.74 cm^3^ ± 3.4 cm^3^ for the right maxillary sinus via manual and automatic analyses, respectively. The left maxillary sinus values were 11.8 cm^3^ ± 4.1 cm^3^ and 11.9 cm^3^ ± 3.2 cm^3^. These results are similar to Prabhat *et al.*'s findings of 11.61 cm^3^ and 10.91 cm^3^ for the right and left maxillary sinuses, respectively. However, our values were lower than those reported by Farias Gomes *et al.*, who found MSV values of 15.2 cm^3^ and 15.3 cm^3^ using ITK-SNAP version 3.0. Statistical analysis revealed a highly significant difference between male and female MSV values for both manual and automatic methods (p-value = 0.001), with males exhibiting larger MSV values. This finding is consistent with several studies, including those by Farias-Gomes, Prabhat, and others. However, Saccucci *et al.* and Chaurasia & Katheriya did not find a significant difference in MSV values between genders. Rani *et al.* identified a gender difference only in the left MSV using MRI. Overall, the gender prediction accuracy (GPA) based on manual calculation of MSV values was 69.6% for the right and 73.9% for the left maxillary sinus. Female predictions were more accurate (73.3% and 80%, respectively) compared to male predictions (62.5% and 62.5%). These results are comparable to those reported by Sharma *et al.* and Fernandes *et al.*, though Fernandes and others used morphometric sinus parameters rather than volumetric analysis. The GPA based on automatic segmentation was slightly lower, at 65.2% for the right and 73.2% for the left maxillary sinus, with female predictions being more accurate than male. These findings were closer to the GPA reported by Sharma *et al.* and Uthman *et al.* though lower than Prabhat *et al.*, who achieved an overall GPA of 83.3%. Our study utilized CBCT for analyzing maxillary sinus anatomy bilaterally, whereas other studies have used MRI or CT. The use of segmentative software in sinus volumetric assessment began with Spaeth *et al.* (1997) [13] and Fernandez *et al.* (2000) [14]. Sahlstrand-Johnson *et al.* [15] pioneered the combined use of manual and automatic methods for MSV estimation, noting that manual methods were less time-consuming, a finding we could not corroborate. Limitations of our study include a small sample size and potential pigment dispersion into adjoining areas.

## Conclusion:

The male patients in our study showed increased right & left maxillary sinus volumes compared to the same in their female counterparts thereby leading to significant statistical difference in the final values of volumes of maxillary sinuses on the basis of both manual & automatic volume calculation. Overall gender prediction accuracy on the basis of manual calculation method for right & left maxillary sinuses were 69.6% & 73.9% respectively while the same on the basis of automatic calculation were 65.2% & 73.9% respectively. Sinus volumetric analysis can be used to predict gender which makes it an indispensable tool in forensic dentistry.

## Figures and Tables

**Figure 1:**
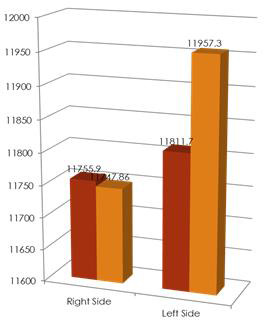
Intergroup comparison of sinus volumes between manual and automatic method in females

**Figure 2:**
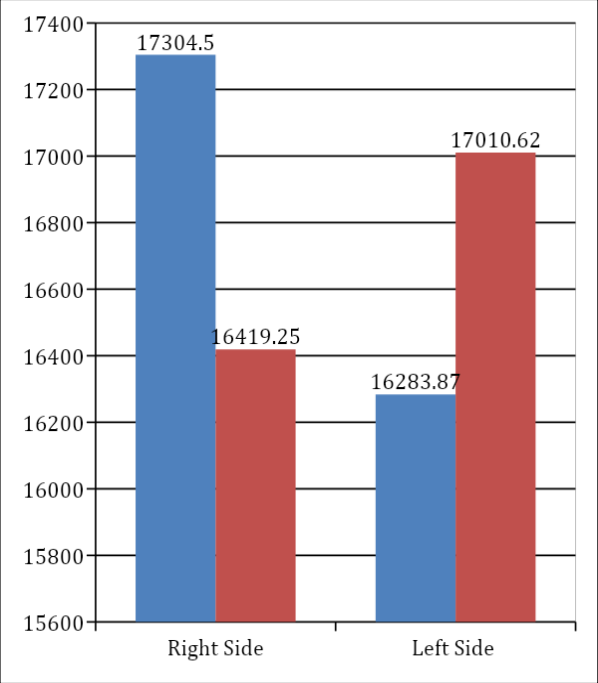
Intergroup comparison of sinus volumes between manual and automatic method in males

**Figure 3:**
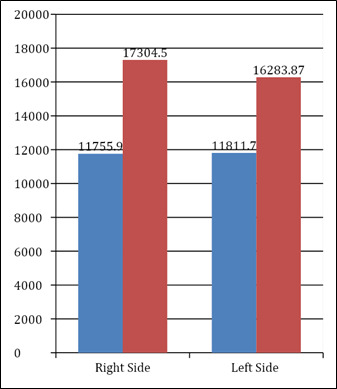
Intergroup comparison of sinus volumes between males and females in manual method

**Figure 4:**
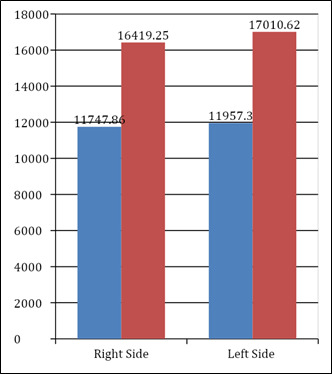
Comparison of sinus volumes between males and females in automatic method

**Table 1:** Intergroup comparison of sinus volumes between manual and automatic method in females

		**Mean**	**Std. Dev**	**Std. Error**	**p value**	**Significance**
Right Maxillary Sinus Volume	Manual	11755.9	4237.16	773.59	0.991	Non-Significant
	Automatic	11747.9	3442.38	628.49		
Left Maxillary Sinus Volume	Manual	11811.7	4176.81	762.57	0.894	Non-Significant
	Automatic	11957.3	3278.78	598.62		

**Table 2:** Intergroup comparison of sinus volumes between manual and automatic method in males

		**Mean**	**Std. Dev**	**Std. Error**	**p value**	**Significance**
Right Maxillary Sinus Volume	Manual	17304.5	7418.69	1854.67	0.725	Non-Significant
	Automatic	16419.3	6659.95	1664.98		
Left Maxillary Sinus Volume	Manual	16283.9	4176.81	762.57	0.778	Non-Significant
	Automatic	17010.6	3278.78	598.62		

**Table 3:** Intergroup comparison of sinus volumes between males and females in manual method

		**Mean**	**Std. Dev**	**Std. Error**	**p value**	**Significance**
Right Maxillary Sinus Volume	Female	11755.9	4237.16	773.59	0.001	Highly Significant
	Male	17304.5	7418.69	1854.67		
Left Maxillary Sinus Volume	Female	11811.7	4176.81	762.57	0.001	Highly Significant
	Male	16283.9	4176.81	762.57		

**Table 4:** Intergroup comparison of sinus volumes between males and females in automatic method

		**Mean**	**Std. Dev**	**Std. Error**	**p value**	**Significance**
Right Maxillary Sinus Volume	Male	11747.9	3442.38	628.49	0.001	Highly
	Female	16419.3	6659.95	1664.98		Significant
Left Maxillary Sinus Volume	Male	11957.3	3278.78	598.62	0.001	Highly
	Female	17010.6	3278.78	598.62		Significant

**Table 5:** Abbreviations: Pf-predicted female, Pm-Predicted male, CP-Correctly predicted, IP-incorrectly predicted

	**LEFT MS**		**RIGHT MS**	
	**Gender prediction accuracy**		**Gender prediction accuracy**	
Original Male (n=80)	Pm <MANUAL>	Pm <AUTOMATIC>	Pm <MANUAL>	Pm <AUTOMATIC>
	50(62.5%)- (CP)	45(56.2%)(CP)	50(62.5%)(CP)	40(50%)(CP)
	Pf <MANUAL>	Pf <AUTOMATIC>	Pf <MANUAL>	Pf <AUTOMATIC>
	30(37.5%)- (IP)	35(43.8%)(IP)	30(37.5%)(IP)	40(50%)(IP)
Original female (n=80)	Pf<MANUAL>	Pf<AUTOMATIC>	Pf <MANUAL>	Pf <AUTOMATIC>
	64(80%)(CP)	67(83.3%)(CP)	59(73.3%)(CP)	59(73.3%)(CP)
	Pm<MANUAL>	Pm<AUTOMATIC>	Pm <MANUAL>	Pm <AUTOMATIC>
	16(20%)(IP)	13(16.7%)(IP)	21(26.7%)(IP)	21(26.7%)(IP)
